# Supercritical Impregnation of Mango Leaf Extract into PLA 3D-Printed Devices and Evaluation of Their Biocompatibility with Endothelial Cell Cultures

**DOI:** 10.3390/polym14132706

**Published:** 2022-07-01

**Authors:** Pilar Grosso, Cristina Cejudo, Ismael Sánchez-Gomar, Mª Carmen Durán-Ruiz, Rafael Moreno-Luna, Lourdes Casas, Clara Pereyra, Casimiro Mantell

**Affiliations:** 1 Chemical Engineering and Food Technology Department, Wine and Agrifood Research Institute (IVAGRO), University of Cadiz, Puerto Real, 11519 Cadiz, Spain; pilar.grossorodriguez@alum.uca.es (P.G.); lourdes.casas@uca.es (L.C.); clara.pereyra@uca.es (C.P.); casimiro.mantell@uca.es (C.M.); 2 Biomedicine, Biotechnology and Public Health Department, University of Cadiz, 11002 Cadiz, Spain; ismael.sanchez@gm.uca.es (I.S.-G.); maricarmen.duran@gm.uca.es (M.C.D.-R.); 3 Institute of Research and Innovation in Biomedical Sciences of Cadiz (INIBICA), 11009 Cadiz, Spain; 4 Laboratory of Neuroinflammation, National Paraplegics Hospital, SESCAM, 45071 Toledo, Spain; rmluna@sescam.jccm.es

**Keywords:** supercritical impregnation, mango leaf, 3D printer, PLA, ECFCs

## Abstract

The addition of natural substances with pharmacoactive properties to polymeric biomedical devices would provide beneficial regarding the assimilation of these endoprostheses when implanted into a patient’s body. The added drug would facilitate endothelization by regulating the inflammatory processes that such interventions entail, preventing contamination hazards and favoring the angiogenesis or formation of blood vessels in the tissue. The present work used mango leaf extract (MLE) obtained through pressurized ethanol for this purpose. Polylactic acid (PLA) in the form of filaments or 3D-printed disks was impregnated by means of supercritical technology with MLE for the culture essays. The release kinetics has been studied and the polymer matrices have been examined by scanning electron microscopy (SEM). The impregnated devices were subjected to in vitro culture of colony-forming endothelial cells. The influence of the different impregnation conditions used for the production of the MLE impregnated polymeric devices on the development of the cell culture was determined by fluorescence microscopy. The best results were obtained from the calcein cultures on 35 °C MLE impregnated into 3D-printed polymer disks.

## 1. Introduction

In recent decades, there has been a growing interest in the use of sustainable and environmentally friendly materials in various fields such as biomedical, pharmaceutical, or food industries. Research on the applications of biodegradable polymers in biomedicine began in the 1960s [1]. Polymeric materials are characterized by being renewable, biocompatible and non-toxic, as well as being susceptible to alterations or additions that modify their functionality and allow simple and economic production. All of this contributes to the fact that biodegradable polymers, such as polylactic acid (PLA), are replacing conventional materials for the production of endoprostheses.

Thanks to the research on biomaterials and the innovations in pharmaceutical technology, a new implant concept allows the introduction of sophisticated therapeutic devices to accomplish controlled and temporary drug delivery, according to drug release profiles and to the corresponding therapeutic targets [2].

These advances are largely attributed to the use of the technique known as 3D printing or additive printing, which was pioneered in the early 1980s by the engineer Charles Hull [3]. This method is based on the layer-by-layer production of three-dimensional drug delivery systems based on digital designs. This method allows customized drug administration devices to be obtained thanks to the possibility of conferring the implants with different external and internal structures and better controlling the percentage of therapeutic agents that they contain [4], as well as the geometric spacing, shape and dosing time. All of this provides the advantage of administering as low and precise doses as required [2]. The flexibility of 3D printing contributes to solving the problem of the individual response to drugs by the different organisms. These features are not met by the conventional production of drugs in batches [4]. In addition, this field goes on to the development of a wide range of 3D printing techniques, such as fused deposition modelling (FDM). Nugroho et al. based their experiment on the study of different parameters and conditions for the optimization of the printing process of dog bone samples by FDM, demonstrating the significant impact of the layer height [5]. On the other hand, through simple ligand exchange procedures, photopolymerization printing is an effective strategy in the fabrication of advanced materials. Zhu et al. claim that it is possible to obtain homogeneous networks of advanced materials, such as inorganic polymers [6]. In addition, Shi et al. developed photopolymerized resins by means of RAFTs, an achievement that opens the door to obtaining advanced composite materials with arbitrary combinations of chemical and mechanical properties [7].

To favor the performance of the polymeric implants for the treatment of diseases and the healing of the damaged tissues, bones and organs, it is necessary to administer substances with pharmacoactive properties. The function of the drug should be to promote endothelization in a controlled manner, facilitating or slowing down the growth of endothelial cells in the prosthesis environment, while avoiding the microbial contamination and inflammation of the surrounding tissue. The growth of cells on the surface of the device is a crucial aspect, since this may have a positive or negative effect depending on the intended use of the device. In the case of a scaffold, a greater endothelization would be positive, while in a stent, it should be more controlled or limited.

Supercritical impregnation is one of the techniques that is arousing most interest in the addition of bioactive substances into polymeric endoprostheses. Impregnation using supercritical fluids reduces the use of toxic solvents and works at moderate temperatures. This enables the use of thermolabile substances such as the polyphenolic compounds that are found in mango leaves. These are bioactive compounds that have exhibited antioxidant, antidiabetic, antihypertensive, anti-inflammatory, and cytotoxic effects against various types of tumors [8]. We have also recently demonstrated that the incubation of endothelial colony-forming cells (ECFCs) with mango leaf extracts enhances the proangiogenic properties of these cells [9].

The main applications of polymeric implants in the biomedical field are cancer treatment, tissue engineering, and bone regeneration. In these cases, the possibility that the implant could be made of a bio-degradable polymer brings certain advantages. If the polymer degrades in a controlled manner and is absorbed by the body, future failures or complications such as infections could be predicted, and it would no longer be an obstacle for future treatments. The degradation of the polymeric material may act as an optimal vehicle for targeted drug or gene therapy. One example is the results obtained with biodegradable poly-L-lactic acid (PLLA) coronary stents in clinical trials with patients undergoing coronary angioplasty. It was concluded that they had the desired safety, efficiency and viability profiles [10].

In a previous work, PLA filaments impregnated with ethanolic mango leaf extract (MLE) with pharmacological properties were generated by supercritical impregnation [11]. In this work, the in vitro activity (antioxidant and anti-inflammatory) of the gene-rated material by supercritical impregnation and subsequent 3D printing was studied, focusing on the supercritical impregnation process. In this sense, the effect of pressure, temperature and amount of extract on the polymer swelling, extract loading, and bioactivity of the final PLA filaments has been analyzed. The results revealed that a polymer with high antioxidant and anti-inflammatory capacities could be obtained. These results were then compared against impregnated PLA samples prior to being 3D printed. The bioactive properties persisted after the printing. These results suggest the feasibility of using functionalized PLA filaments for 3D printing and biomedical applications. In the present work, the biocompatibility through the attachment of endothelial cells to the surfaces of the impregnated PLA devices using mango leaf extracts with pharmacoactive properties has been analyzed. The main novelty of this work is focused on the determination of this biocompatibility using an ex vivo test. In this study, two different conformations have been analyzed: on one hand, PLA surfaces generated by 3D printing, which have been later impregnated with the extract using supercritical technology, and on the other hand, the impregnated PLA filaments and their subsequent use in conventional 3D printers. The effect that such printing has on the biocompatibility of the generated material and on the development of controlled endothelization has been analyzed.

## 2. Materials and Methods

### 2.1. Raw Materials

The natural extract used for the impregnation processes was obtained from dried mango leaves (sp. *Mangifera indica* L.) of the Kent variety, collected in May 2017 and supplied by Institute of Subtropical and Mediterranean Horticulture “La Mayora” (CSIC. Malaga). The mango leaves were dried at room temperature and crushed to reduce their size and favor the contact between the raw material and the solvent used for the extraction process.

PLA (polylactic acid), a biodegradable synthetic polyester, was the polymer selected for the supercritical impregnation, 3D printing and biocompatibility study. It was supplied by the BQ brand of Mundo Reader, SL (Madrid, Spain) in a transparent format and 1.75 mm nominal diameter.

Endothelial cells (ECFC) were used for the biocompatibility study. The ECFCs were provided by Dr. Moreno-Luna, National Paraplegics Hospital, (Toledo, Spain). The cells were isolated from discarded samples of subcutaneous white adipose tissue, following the procedure described by Lin et al. [12].

### 2.2. Enhanced Solvent Extraction (ESE)

**Figure 1:**
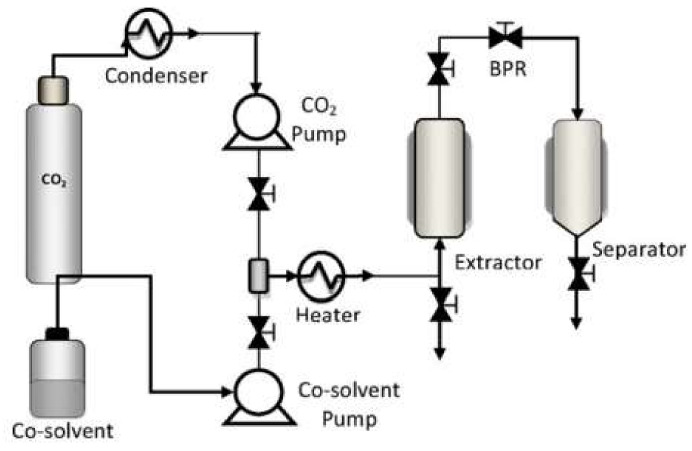
Flow diagram of the supercritical extraction and impregnation equipment.

The mango leaf extracts were obtained by enhanced solvent extraction (ESE). The process was carried out using a Thar Technologies Inc (Pittsburgh, PA, USA) high-pressure extraction plant. A SF1000 model equipped with a 1L volume extractor was employed. The system was fitted with two high-pressure pumps P50 model with double piston to insert both the CO_2_ and the liquid solvent into the extractor. A heat exchanger upstream of the pump cooled the carbon dioxide by means of a cryostat bath (Fisher Scientific, Waltham, Massachusetts, EE.UU.) down to below 5 °C, ensuring its transition from gaseous into liquid phase. After going through the pump, a high-pressure heat exchanger preheated the solvent back to the desired operating temperature. A back pressure regulator (BPR), configured to control and maintain the operating pressure inside the extractor, was located at the outlet of the extractor. A flow diagram of the system used is shown in Figure 1.

Polyphenolic compounds are the most active compounds in mango leaves. The enhanced liquid extraction technique is characterized by using a mixture of carbon dioxide and a liquid solvent, in our case, ethanol at a high ratio. CO_2_ modifies the diffusing properties of the liquid solvent and facilitates its penetration capacity, which improves the efficiency of the process. Hence, the extraction was carried out using a CO_2_:ethanol mixture.

The crushed mango leaves samples were wrapped into filter paper cartridges, containing about 200 g of the raw material. The process was carried out in batches using 20 h extraction time and an initial amount of ethanol of approximately 450 mL. The pressure and temperature conditions were 200 bar and 80 °C, respectively.

#### 2.2.1. Determining the Antioxidant Activity

The bioactivity of the mango leaf extract was determined by means of the DPPH method. This technique allows us to determine the antioxidant capacity of foods and synthetic compounds, based on the reduction of the free radical 2,2-Diphenyl-1-picrylhydrazyl (DPPH). This species, characterized by the presence of unpaired electrons in its external orbital, is susceptible to interacting with compounds that present antioxidant activity through the cession of a hydrogen atom [13]. The reduction of this compound leads to a change from purple to yellow color, with the subsequent drop of absorbance, which is detected spectrophotometrically at 515 nm maximum absorbance.

Firstly, it was necessary to determine the inhibition percentage range, for which seven dilutions of mango leaf extract in ethanol were prepared within a 50–2500 ppm concentration range. The measurement blank corresponded to pure ethanol, which presents 0% inhibition. A stock solution of DPPH (Sigma-Aldrich (Steinheim, Germany) in milliQ water (6 × 10^−5^ M) was also prepared. Then, 1 mL of DPPH dilution was added for every 3.9 mL of mango leaf extract dilution. The absorbance was measured after 0, 1 and 2 h by means of a Cary 60 UV–Vis spectrophotometer by Agilent Technologies (Santa Clara, CA, USA). The DPPH inhibition and remaining percentages at steady state were considered to be the indicative measures of the antioxidant capacity of the mango leaf extract:

(1) $$\%I=\frac{Abs_{0}-Abs_{f}}{Abs_{f}}\cdot 100$$

(2) $$DPPH\;remaining\;percentage\;\left(\%\right)=100-\%I$$

The antioxidant activity was experimentally determined by calculating the EC50 parameter, which represents the effective extract concentration required to reach 50% of the antioxidant activity according to DPPH inhibition. The antioxidant activity index (AAI) gives us a comparative view of the antioxidant capacity of the different samples:

(3) $$EC_{50}=-1.3733x+91.123$$

(4) $$AAI=\frac{{\left[\;\right]}_{DPPH}\;\left(\mathrm{mg}/\mathrm{mL}\right)}{EC_{50}}$$

#### 2.2.2. High Performance Liquid Chromatography (HPLC)

This technique was applied to the characterization of the major polyphenols present in the extract. An Agilent Technologies Series 1100 chromatograph equipped with a UV–Vis detector and an automatic injector was the equipment used for this purpose.

ChemStation^®^ HP was the software solution used for data analysis. The stationary phase used was a Synergi Hydro-RP C18 column (150 mm × 3 mm i.d., 4 μm) (Phenomenex, Torrance, CA, USA) with 4.0 mm × 2.0 mm i.d. The mobile phase consists of water/0.1% formic acid (solvent A) and acetonitrile/0.1% formic acid (solvent B). A detailed description of the procedure as well as its quantification was described in a previous work [14].

### 2.3. Supercritical Impregnation (SCI)

The supercritical fluid impregnation processes of the mango leaf extract onto PLA were carried out in high-pressure equipment by Thar Technologies Inc. (Pittsburgh, PA, USA). The equipment, operation and commissioning were consistent with those described previously in Section 2.2.

To analyze the effect of 3D printing on the biocompatibility of the generated material, two different conformations of PLA were impregnated with mango leaf extract:

On the one hand, the PLA original filaments were impregnated to be later fed into a 3D printer. That is, the impregnated filament was used to print the disks that would later be analyzed. The impregnation, in this case, was carried out using the same SF1000 model system described in Section 2.2. The final material obtained will be referred to as SCI + 3D hereinafter.

On the other hand, disks with the same conformation as the previous ones were generated by 3D printing. This time, they were generated using non-impregnated PLA filaments. Subsequently, the devices were subjected to supercritical impregnation. In this case, the impregnation was carried out using an SF100 model system, which differs from the previous one in that it is fitted with a 100 mL impregnation cell. The resulting product will hereinafter be referred to as 3D + SCI.

**Figure 2:**
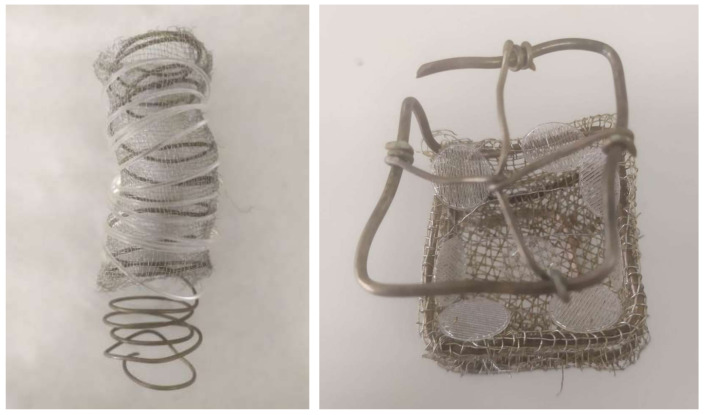
Polymer metal supports used over the impregnation process.

For both conformations, a volume of extract dissolved in ethanol was added at 0.0782 g/mL concentration, which corresponds to 3% of the reactor’s total volume. For the impregnation of the PLA filaments, 30 mL of mango leaf extract was introduced in the vessel, while for the impregnation of the disks, only 3 mL were used. Approximately a 2.10 mL filament fragment was used for each impregnation procedure, while the disks were treated in 7- or 8-unit batches, as shown in Figure 2.

To make it easier for the impregnation process to develop correctly and for the extract to be distributed in the most efficient and homogeneous way throughout the surface of the filaments and disks, these were attached to customized metal supports before they were impregnated. The role of these supports was to prevent any contact between the disks or between the filament and the vessel walls.

The impregnation process was carried out in batch mode for 2 h, using only pure CO_2_ as solvent. Therefore, only one high-pressure pump was employed. The impregnation conditions used for the tests were based on previous works by our research team [11]. Table 1 shows the four different conditions used for the impregnation tests.

The BPR was configured to control the depressurization of the system after the impregnation time had elapsed. The depressurization rate for conditions 1 and 3 was 40 bar/min, and for conditions 2 and 4 was 100 bar/min.

Finally, the diameters of the filaments impregnated under the different conditions were measured to determine the swelling of the PLA that had been in contact with the supercritical fluid.

### 2.4. 3D Printing

**Figure 3:**
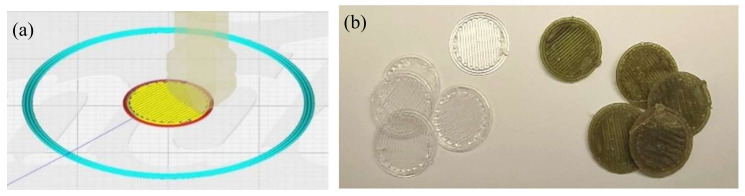
The 3D virtual image (**a**) and photographs (**b**) of freshly printed disks and after being used for the cell proliferation assays.

An ANYCUBIC Mega S model 3D printer was used to produce both types of disks. In Figure 3 are shown both the virtual model (3a) and the obtained disks after printing (3b). The main parameters and printing conditions applied to generate the disks were set up using the application “Fusion360” (Autodesk) and are shown in Table 2 below:

### 2.5. Release Kinetics Study of Mango Leaf Extract

The release kinetics of the mango leaf extract impregnated into the different polymer conformations was examined to determine and control the effect of the different bioactive substance levels on the ECFCs cultures. This aspect must be rigorously studied, since an excessive release of the extract could lead to toxicity problems that would affect the cell cultures instead of benefiting from the pharmacoactive properties of the extract, which would be counterproductive regarding the production of endoprostheses or any other biomedical devices.

In the first place, it was necessary to construct a calibration line relating the concentration of the extract in the medium to its corresponding absorbance levels. Two mL of extract were concentrated for their subsequent dilution into 10 mL of phosphate-buffered saline (PBS), achieving a concentration of 94850 ppm. Then, dilutions at 3 ppm, 5 ppm, 20 ppm, 30 ppm, 55 ppm and 60 ppm were performed. The absorbance corresponding to each of the dilutions were determined by means of a spectrophotometer at 275 nm, which refers to the maximum value of the mango leaf extract spectrum according to previous calculations.

(5) Abs=0.0156C

Once the calibration line was constructed, the impregnated polymer samples were submerged in PBS, and the supernatant liquid was measured during the first 6 h. The blank was made up of a non-impregnated PLA disk immersed into 4 mL of PBS. Four different impregnation conditions were performed on two disk types, which are those that are 3D printed and then impregnated (3D + SCI) and those that are 3D printed using a pre-impregnated filament (SCI + 3D). This experiment was designed to examine the release of the extract in a limited time range in order to ensure the reliability and robustness of the results obtained. All experiments were conducted in duplicate. After the first hours of release, some variations or alterations in the absorbance levels could be observed, associated both with the deterioration of the released products and, above all, with a high degradation of the control disk with respect to the impregnated ones, due to the instability of the polymer when in direct contact with a slightly basic pH solution (≈7.4).

### 2.6. Scanning Electron Microscopy (SEM)

A Nova NanoSEM 450 scanning electron microscope from the Central Services for Science and Technology Research at the University of Cadiz (Spain), was used to analyze the surface and polymer matrix of the impregnated PLA disks under the different conditions and impregnation modes used. For this purpose, the samples were sputtered with a 10 µm gold layer using a Cressington Sputter Coater 208 HR [11].

### 2.7. Endothelial Cell Culture on PLA Disks

Endothelial colony-forming cells (ECFCs) were isolated from human white adipose tissue, as previously described [12], and cultured in 1% gelatin (Sigma-Aldrich, Steinheim, Germany) precoated plates using EBM-2 medium (Lonza, Morrisville, NC, EE.UU.) supplemented with 10% Fetal bovine serum (FBS), 1× Penicillin/Streptomicin (P/S) and specific growth factors, except for hydrocortisone (Lonza, Morrisville, NC, EE.UU.) [13]. The ECFCs were cultured at 37 °C and 5% CO_2_ until reaching 90% confluence. The ECFCs between passages 5 and 7 were used for all the experiments.

Four control (non-impregnated) disks were deposited together with three replicates corresponding to each of the four impregnation conditions studied in two 24-well plates. One plate contained the disks produced using impregnated PLA (SCI + 3D), while the other contained those disks that were impregnated after the printing process (3D + SCI). The disks were treated with gelatin and then washed off with 1× phosphate buffered saline (PBS). The cells were seeded on the disks and incubated in EBM-2 culture medium for 72 h, inside the CO_2_ incubator, with a concentration in CO_2_ of 5% and at 37 °C.

### 2.8. Cell Viability and Morphology Analysis

For the viability study and the monitoring of the cell cultures on the PLA disks impregnated with mango extract, a treatment with calcein was performed to allow the visualization by means of an Olympus IX81 fluorescence microscope (Barcelona, Spain) of the endothelial cells that had adhered to the surface of the polymer. The disks were washed off using PBS and incubated for 1 h at 37 °C with 1 mM calcein (Sigma-Aldrich, Steinheim, Germany) diluted to 4 µM in 500 µL of PBS per well. Several photomicrographs were taken at 4× magnification covering four different fields on each disc, in addition to capturing images at 10× to visualize and characterize the morphology of the adhered cells.

### 2.9. Statistical Analysis

Representation and analysis of the data from the cell culture study was performed using the application GraphPad Prism 9. A Shapiro–Wilk test was used to determine whether the data followed a normal distribution. Subsequently, the homogeneity of the variances was confirmed using Levene’s test, and ANOVA for multivariate analysis was used for the comparison of the means parametric tests followed by post hoc tests for pairwise comparison using Tukey’s test. The data were represented as mean ± SEM (standard error of the mean) and were considered statistically significant when the *p*-value was < 0.05.

## 3. Results and Discussion

### 3.1. Enhanced Solvent Extraction (ESE)

Natural extracts of *Mangifera indica* L. are obtained, both at laboratory and industrial scale, by traditional extraction methods [15,16]. However, in recent decades, the food, cosmetic and pharmaceutical industries have increased their safety requirements to protect the health of consumers and the environment, so that more efficient and environmentally friendly processes are demanded. In the present work, the mango extracts were obtained by means of ESE. The ESE method consists of adding high proportions of polar co-solvents to CO_2_. This technique has proven to be an efficient strategy to overcome the problem of low recovery and has been successfully applied to the extraction of polar solutes such as the phenolic compounds present in mango leaves [17].

The extracts obtained by ESE using a mixture of CO_2_ + 50% ethanol were characterized prior to the impregnation study. The overall yield of the process (10.7%) was high, obtaining an extract with a concentration of 78200 ppm (Table 3). High temperatures enhance the mass transfer of the analytes into the solvent, and this results in a faster and more efficient extraction process. Pressure also favors analyte-solvent contact as it reduces the surface tension of the solvent, increases the penetration of the solvent into the matrix pores and improves the control of the issues associated with air bubbles [18].

With respect to its antioxidant activity, the mango leaf extract presented values of EC50 = 29.945 ppm and AAI = 0.7703 (Table 3). This is consistent with the result of the extracts obtained from the same raw material using pressurized liquid extraction through a hydroalcoholic mixture (50:50 water: ethanol) [19]. It was concluded from these results that the mango leaf extract obtained had a moderate antioxidant activity, which could provide some benefits apart from any other potential pharmacoactive properties deriving from the application of the extract, which would remain to be studied.

In a previous publication by the same authors, some of the major polyphenols in mango leaves, i.e., mangiferin, gallic acid, and iriflophenone 3-C-β-D-glucoside, were studied [17]. Therefore, the extract was chemically characterized in terms of these phenolic compounds. The results as presented in Table 3 revealed that iriflophenone 3-C-β-D-glucoside was the predominant compound in the ethanolic extract followed by mangiferin and gallic acid. The results from the chemical characterization of the extract confirmed that it is suitable to be used for the impregnation of polymers and confer them with biomedical properties.

### 3.2. Supercritical Impregnation (SCI)

**Figure 4:**
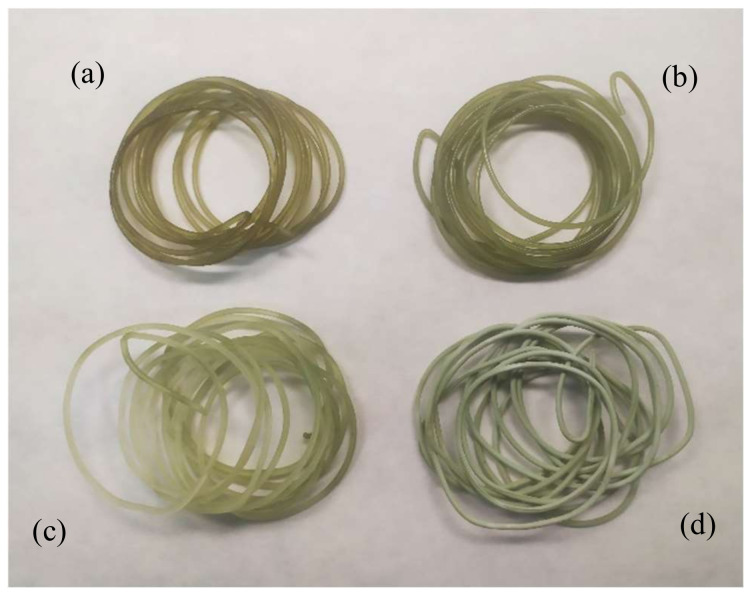
PLA filaments impregnated with MLE: (**a**) 35 °C, 100 bar; (**b**) 35 °C, 400 bar; (**c**) 55 °C, 400 bar; and (**d**) 55 °C, 100 bar.

In order to compare the effect of pressure, temperature and 3D printing on the biocompatibility of the polymeric devices with endothelial cells, the impregnation process was performed under two sets of pressure and temperature conditions. The PLA filaments to be later used for 3D printing and the PLA 3D-printed disks were impregnated. Figure 4 shows the filaments impregnated (SCI + 3D) with mango leaf extract using an ethanolic base under the four supercritical conditions analyzed (100 and 400 bar, at 35 and 55 °C). The effect of pressure and temperature on the impregnation process was already discussed in a previous work developed by our research group [11]. In that work, it was concluded that under low-pressure values (100 bar), the loading seemed to be favored by the temperature, i.e., at a 100 bar constant pressure. When the temperature was increased, the density of the solvent was reduced, which resulted in an increment of the solute concentration in supercritical phase, and therefore an enhanced impregnation with increasing temperature (Figure 4a,d). However, when the pressure used was 400 bar, the higher density of the carbon dioxide caused a higher affinity of the compounds with the supercritical phase, which resulted in a lower impregnation. These results, which have been described in our previous work, agree also with those reported by other authors [20,21]. Figure 4 shows a variation in the green color intensity of the impregnated polymer, which is indicative of the effect that pressure and temperature have on the impregnation process. Thus, the lowest color intensity corresponds to the highest pressure (Figure 4c), which indicates a poorer impregnation. In addition, a change in hue can be observed in the image, especially in Figure 4d, that corresponds to impregnation achieved at the highest temperature studied.

On the other hand, a variation in the opacity (Figure 4d) of the samples has also been observed, which is indicative of the swelling effect that carbon dioxide has on this polymer. This effect on the polymer can be seen in Table 4, where the impregnated filament diameter values are listed along with their diameter increasing percentage. This increment in diameter is associated with the effect known as foaming. Foaming is a process that arises when a polymer is brought into contact with supercritical CO_2_, since it diffuses among the polymer chains and turns the polymer into a plastic state by lowering its vitreous transition temperature. This system reaches a supersaturated state that causes phase separation and the formation of pores within the polymer matrix. This technique is mainly applied to amorphous polymers, but also it can be also applied to polymers with a high crystallinity or vitreous transition temperature [22]. Since these filaments are to be used for 3D printing, it is desirable that the polymer does not undergo a high diameter increment. According to the results from previous studies, at 35 °C, the percentage of diameter increment is relatively low compared to its increment when the tests are performed at 55 °C. While neither of the two pressure levels causes any significant differences at 35 °C, when the impregnation is performed at 55 °C and 400 bar (most extreme conditions), a significant diameter increment can be observed. All the filament samples subjected to these conditions suffered structural damages, and the polymer’s integrity was altered.

Once the MLE impregnated/printed polymeric material had been produced, it was necessary to determine the capacity of the material to release the pharmacoactive substance into the medium, since the polymers undergo various modifications in their structure and surface due to the impregnation and printing processes. In fact, the level of porosity will be different depending on the order in which the impregnation and printing processes are carried out. When the filament is impregnated, pores are created in its structure as a consequence of getting in contact with CO_2_. This porosity could disappear again during the printing process, since the polymer melts at 200 °C so that it can be deposited through the nozzle according to the target design. On the other hand, when the polymer is impregnated after printing, the porosity of its internal structure will be greater. These facts directly affect the internal structure of the polymer and, therefore, will affect the distribution and release of the impregnating mango leaf extract. The degree of porosity on the surface of the polymer is a very important variable to be controlled, since a greater porosity will imply a greater surface area available for both the impregnation of the extract and the subsequent adhesion and growth of the cells.

The release of a drug follows a curve where several stages can be distinguished. The initial stage is often described as a burst release, and it is a diffusion-governed phase. In this stage, a large amount of the drug mass is transferred over the first moments of contact with the release medium, due to the high diffusion rate of the molecules that are located on the outermost surface of the polymeric structure or in the pores that are directly adjacent to its surface. It is very important to determine this first stage in which the release is exclusively by diffusion and the polymer has not yet started its degradation, since it allows us to determine the suitability of the polymeric material for its use in biomedicine.

**Figure 5:**
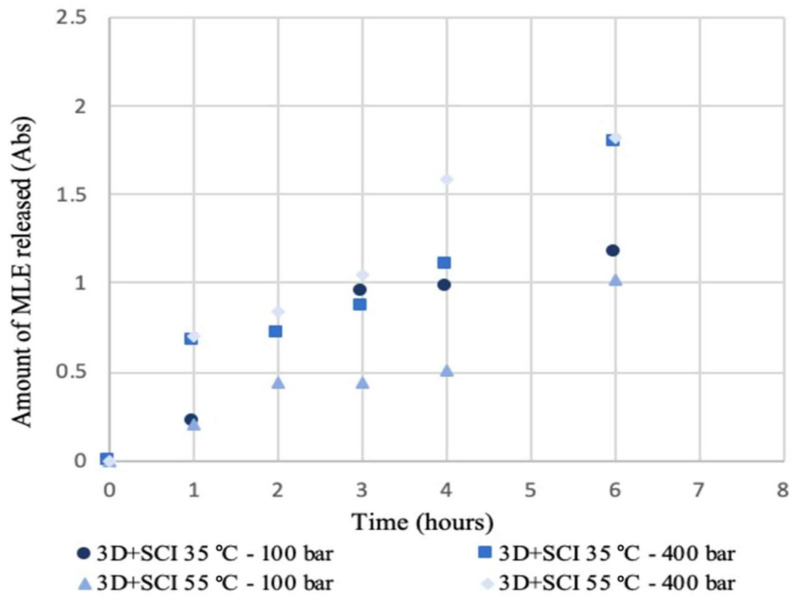
Release kinetics of 3D + SCI samples produced under different pressure and temperature conditions.

**Figure 6:**
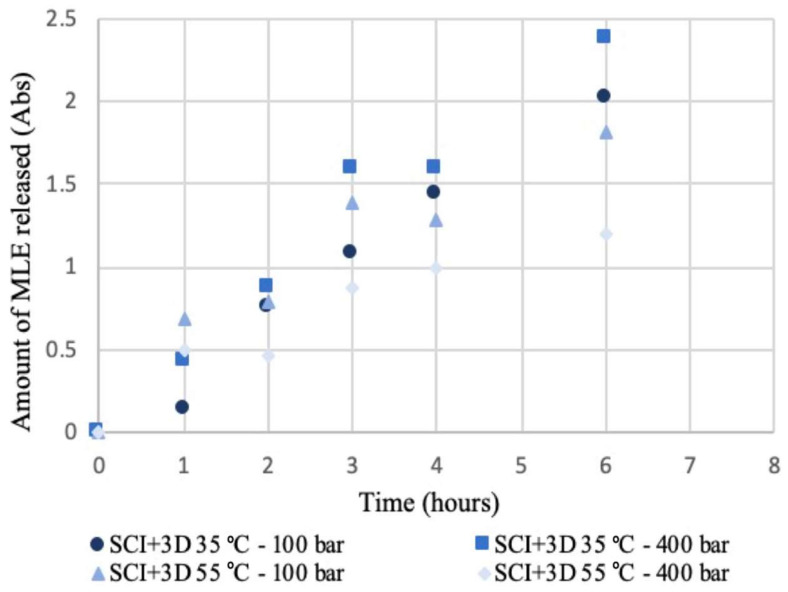
Release kinetics of SCI + 3D samples produced under different pressure and temperature conditions.

Figure 5 and Figure 6 show the respective release profiles of mango leaf extract from 3D + SCI and SCI + 3D disks into PBS, at the initial stages of the process. Although the trends are not very clear, it can be observed that the release rate seems to be faster and linear for SCI + 3D disks, while 3D + SCI disks generally release a lower amount of extract during this stage. It can therefore be concluded, first of all, that during the first hours in contact with the release medium, a higher release from the SCI + 3D disks is observed, and this trend decreases as the hours elapse (Figure 5). The opposite trend can be observed from the data collected in Figure 6. This behavior can be explained by the dissimilar porosity of the samples after the 3D printing process. The printing process decreases the porosity of the polymer hindering the diffusion of the drug.

On the other hand, the influence from the pressure and temperature on the release profiles used during the impregnation process should be evaluated. From the data collected in Figure 5, it can be observed that in general, the samples produced under 400 bar at both 35 and 55 °C present a greater release of extract respecting to the samples produced at 100 bar. These data agree with the trend observed in Figure 6 on the samples produced at 35 °C, which exhibited the greatest amounts of extract released. However, the behavior of the samples produced at 35 and 55 °C under 100 bar present a greater similarity, being less disperse. The same trend had been revealed by both impregnation configurations in a previous work [11]. In addition, the results are in agreement with that obtained by other authors in the supercritical impregnation of carvone from PLA films [23]. In this study, a straight trend is obtained in the first instants of the release, and the slope becomes less pronounced when the density of the supercritical carbon dioxide is increased. On the other hand, in the analysis of the release ketoprofen impregnated in PLA [24], a straight line is observed in the initial stages (phase I), describing likewise a release in burst.

### 3.3. Cell Viability

In order to evaluate the viability of ECFCs cultured on disks impregnated with mango leaf extracts, the cells were cultured for 72 h and their adhesion and distribution were determined by cell labeling with calcein (green dots), which allowed their counting per unit area (mm^2^) by fluorescence microscopy.

**Figure 7:**
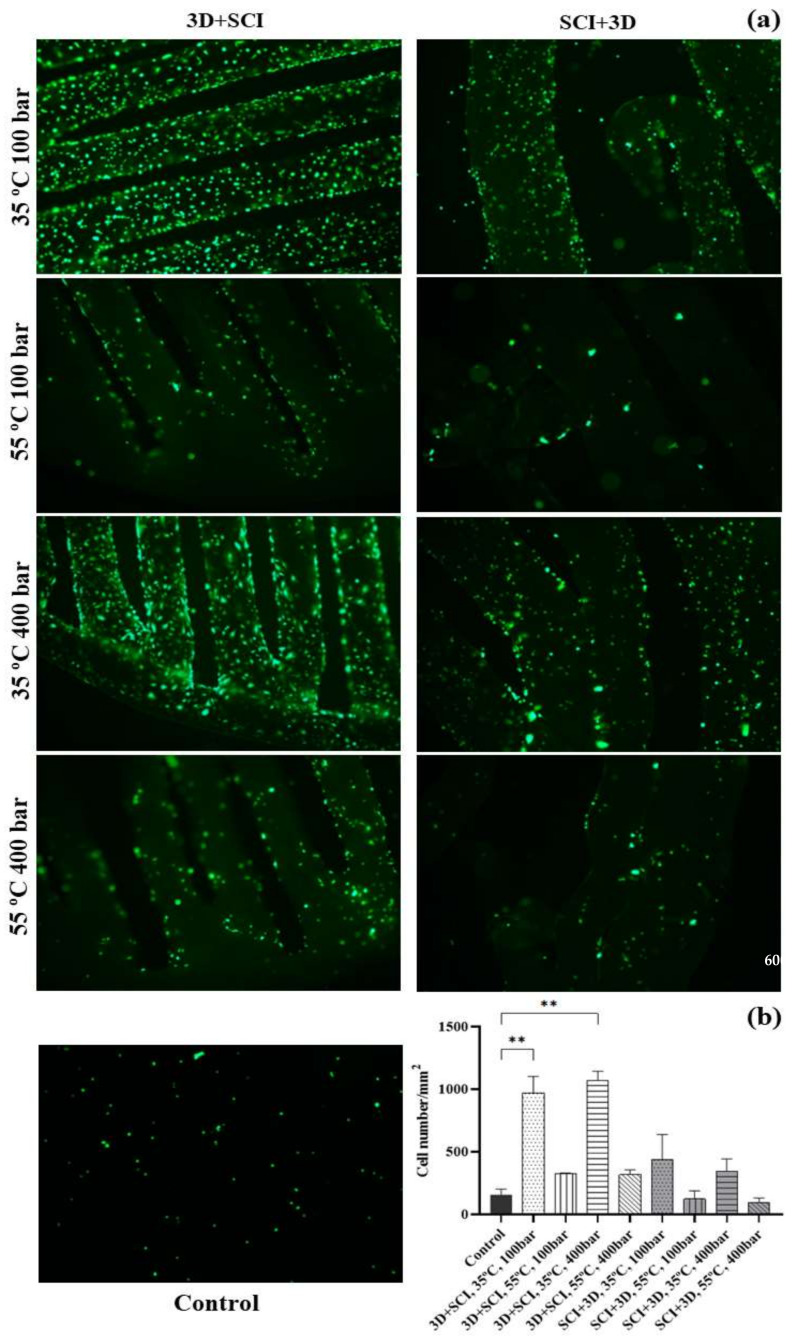
Viability of ECFCs grown on MLE impregnated PLA disks. (**a**) Representative images of ECFCs cultured on PLA disks impregnated with mango extracts under different sets of conditions, compared against a non-impregnated control disc. Calcein labeling can be observed (green dots) after incubation for 1 h at 37 °C. The photographs were taken at 4× magnification. (**b**) Graphical representation of the cell count per unit area under the different impregnating conditions of the PLA disks. The values are represented as mean ± SEM (*n* = 3). ** *p*-value < 0.01.

A large difference was observed in the number of cells present in the control disks with respect to that on the disks impregnated under different conditions (Figure 7). It should be highlighted the higher number of cells observed on the disks that had been printed prior to their impregnation (3D + SCI) at 35 °C with respect to the rest of the disks. When 400 bar pressure was used, a small increase in the number of cells was observed with respect to those produced under 100 bar. However, it could be noticed that the distribution of the cells throughout the polymer surface was more uniform and complete on the disks impregnated at 100 bar. Both sets of conditions presented statistically significant differences with respect to the control disks in terms of cell count (Figure 7).

A noticeable difference was observed when the operating conditions reached 55 °C, with a significant decrease in the number of adhered cells. At this temperature, the disks undergo some deformation and do not maintain their original flat conformation. As the disk surface becomes wavier, cell adhesion may be hindered, and cells may concentrate in the valley-shaped areas, as can be assumed by the distribution of cells limited to certain areas. On the other hand, it was observed that under these conditions the polymers underwent the phenomenon known as foaming [25], which affects the pattern in which the extract is impregnated into the polymer and, therefore, the way it is released into the medium.

Finally, a drastic change takes place when the polymer is impregnated before passing through the 3D printer. Producing printed disks using these filaments was particularly difficult, since they generated very irregular and unstable surfaces. On the other hand, it is worth noting that as explained in previous sections, the porosity of this type of disks is significantly lower than that of disks that are first printed and then impregnated, which results in a poorer release of the extract. These disks impregnated at 55 °C provided the worst culture conditions for ECFCs, since not only do they have a more irregular structure, but also, because of the foaming phenomenon, a poorer extract release can be observed.

**Figure 8:**
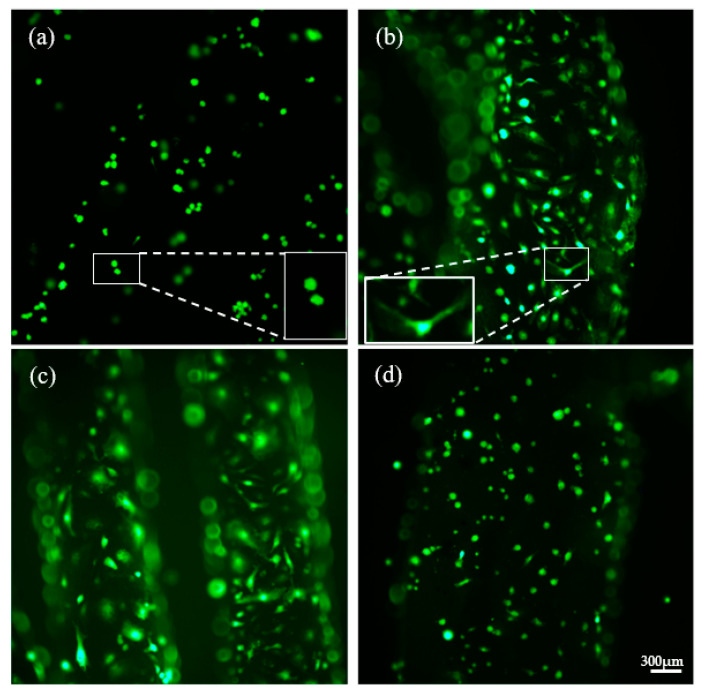
Analysis of the cell morphology of the ECFCs cultured on PLA disks impregnated with mango extract. Images of the ECFCs cultures on PLA disks impregnated with mango extracts under different conditions and non-impregnated control disc. The photographs were taken at 10× magnification: (**a**) PLA control; (**b**) 35 °C, 100 bar (3D + SCI); (**c**) 35 °C, 400 bar (3D + SCI); (**d**) 35 °C, 400 bar (SCI + 3D).

In addition to notable differences in the number of cells, it was also observed that cell morphology also differed according to the different conditions. Figure 8 shows the images taken at 10×. They are representative of the different morphologies observed in the cell growth assays on PLA disks impregnated under different conditions.

A reduced number of cells with a spherical morphology could be observed on the PLA control disks (Figure 8a). This is associated with a non-favorable physiological state of the cells, and suggests that they might be subjected to certain stressing conditions corresponding to an unsuitable environment for their proliferation, growth and differentiation. This state would eventually lead to the senescence of the cells, where they lose their original conformation and become contracted and rounded [26,27]. As a result of the impregnation with mango leaf extract, the number of cells on the surface of the disks increased notably, and so did the quality of the culture. Figure 8b,c impregnated at 35 °C and 100 bar and 400 bar, respectively, show that the endothelial cells present a more elongated and polygonal morphology, which is characteristic of healthy endothelial cells [28]. This cell morphology and distribution suggests that the coating of the polymers with the extract provides optimal conditions for the growth and maintenance of the culture. This may be due to the pharmacoactive and antioxidant properties of mango leaf extract, which had been previously studied, and to the anti-apoptotic and pro-angiogenic properties that mango leaf extract exhibit regarding the culture of ECFCs in vitro [9,29].

The set of conditions 35 °C and 400 bar for SCI + 3D disks are shown in Figure 8d. The difference between the two types of disks is obvious. Despite the presence of mango leaf extract, the morphology of the cells in this case corresponds almost entirely to that of the control cells. This may lead us to think that the fact of impregnating the filaments prior to printing could deteriorate the pharmacoactive properties of the extract. This occurs when the antioxidant activity associated with polyphenolic compounds is reduced due to exposure to high temperatures [30] during the printing process, together with the modification of the internal structure of the polymer and the releasing profile. The rounded morphology of the cells on the control disks was also present in the disks whose impregnation process had been carried out at 55 °C, either at 100 or 400 bar.

### 3.4. Scanning Electron Microscopy (SEM)

Those samples that exhibited the most promising and representative results from the cell culture assays were selected to be observed by scanning electron microscopy to understand the differences in the polymer surface responsible for the different degree of coating and cell viability. The selected samples were first printed and then impregnated (3D + SCI) at 35 °C at either 100 or 400 bar.

**Figure 9:**
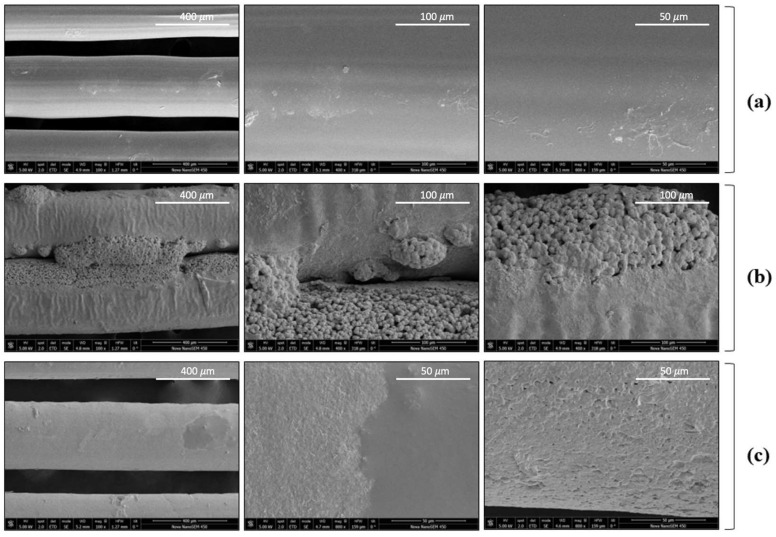
Scanning electron microscopy examination of 3D + SCI samples: (**a**) PLA control disks (100×, 400×, 800×); (**b**) 35 °C, 100 bar (100×, 400×, 400×); and (**c**) 35 °C, 400 bar (100×, 800×, 800×).

The PLA control disks (non-impregnated) (Figure 9a) were characterized by an almost uniform and smooth surface. Hardly any irregularities could be observed, which could be inherent to the original material or could have been the result of the 3D printing process.

However, it could be confirmed from the images that the surface and the internal polymeric matrix were affected by the impregnation process, both under 100 and 400 bar. The surfaces had undergone major modifications and now presented roughness and a higher degree of porosity with respect to the control sample, as an effect of being in contact with supercritical CO_2_. These results are in agreement with those obtained in previous studies, where the same raw materials were used [31]. The polymer surface changes are more evident in Figure 9b, which corresponds to the essays conducted at 35 °C and 100 bar, where the mango leaf extract, with a spherical shape, practically covers the surface completely and penetrates inside the polymer. Regarding Figure 9c (35 °C, 400 bar), the surface remained relatively uniform, and no protrusions related to the penetration of the extract into the polymer matrix could be observed, even though a surface coating could be noticed. The differences between Figure 9b,c are attributed to higher MLE loading at 100 bar with respect to 400 bar, as well as a better penetration of the supercritical CO_2_ into the polymer in the presence of the extract, increasing the internal free volume which would be in agreement with results obtained in previous work [11].

## 4. Conclusions

Different conformations have been developed using PLA filaments and supercritical CO_2_ impregnation to later conduct ECFC in vitro assays. For this purpose, the release kinetics of mango leaf extract was previously studied, and it was observed that the two conformations—SCI + 3D and 3D + SCI—of the impregnated PLA disks followed different trends. Thus, the 3D + SCI disks exhibited a slower initial extract release rate, although their profile was more stable. On the other hand, the in vitro viability assays on impregnated disks revealed large differences when compared to the control disks, with poorer culture results. Specially between these and the 3D + SCI disks impregnated at 35 °C and either 100 or 400 bar. The culture viability decreased as the temperature of the impregnation process increased to 55 °C, which could be due to foaming, or to the degradation and inactivation of the antioxidant polyphenolic compounds in the extract when subjected to the high temperatures of the 3D printing process (200 °C). Therefore, the best ECFCs cultures were registered on 3D + SCI disks impregnated at low temperature (35 °C) and either at 100 or 400 bar, reaching values of 1000 cells/mm^2^, which could be further confirmed by cell morphology analysis. These results confirm that MLE supercritical impregnation on PLA devices is a suitable technique to be used in biomedical applications. Future work should be aimed at analyzing the material generated in animal models and at determining the economic viability of the supercritical impregnation process. The cost of pharmacoactive endoprostheses reaches thousands of euros in the market. The possibility of using filaments already impregnated with pharmacoactive substances to generate biomedical material using 3D printing raises interesting expectations for regions of the world with limited access to these materials.

## Figures and Tables

**Table 1:** Impregnation conditions.

	Setup 1	Setup 2	Setup 3	Setup 4
Temperature	35 °C	35 °C	55 °C	55 °C
Pressure	100 bar	400 bar	100 bar	400 bar

**Table 2:** Design parameters and printing conditions.

Layer Height	Thread Thickness		Diameter	Outer Turns
0.2 mm	0.4 mm		1 cm	1
**Filament temperature**		**Bed temperature**		**Printing rate**
200 °C		60 °C		25 mm/s

**Table 3:** Chemical and functional characterization of the extract.

ELE Performance (%)	10.7 ± 1.2
Extract concentration (ppm)	78200 ± 231
EC50 (ppm)	29,945 ± 0.14
AAI	0.7703 ± 0.003
**Majority compounds identified (µg/mL)**	
Gallic acid	312.6 ± 13.4
Mangiferin	601.8 ± 18.5
Iriflophenone 3-C-β-D-glucoside	1403.6 ± 16.4

The results are expressed as the mean value ± standard deviation of the assays carried out in duplicate.

**Table 4:** Diameter of the MLE-impregnated PLA filaments.

	35 °C, 100 bar	35 °C, 400 bar	55 °C, 100 bar	55 °C, 400 bar
**Diameter (mm)**	1.753	1.753	1.778	1.854
**Increment (%)**	0.17	0.17	1.6	5.9

## Data Availability

All the data presented in this study are available in this article.
